# COGNITIVE AGING ACROSS NATIONAL CONTEXTS: EVIDENCE FROM THE HARMONIZED COGNITIVE ASSESSMENT PROTOCOLS

**DOI:** 10.1093/geroni/igac059.408

**Published:** 2022-12-20

**Authors:** Lindsay Kobayashi, Alden Gross, Kenneth Langa

## Abstract

The global burden of dementia is rapidly rising and shifting to low- and middle-income countries. The triangulation of evidence across country contexts is essential for unlocking the causes of dementia and reducing its global burden. The Harmonized Cognitive Assessment Protocol (HCAP) is a recent innovation administered in the US Health and Retirement Study and several of its International Partner Studies. For the very first time, these HCAPs provide high-quality data for cross-national comparisons of later-life cognitive function that are sensitive to linguistic, cultural, and educational differences across diverse country contexts. However, despite the common HCAP protocols, human cognitive function does not lend itself to direct comparison across diverse populations without careful consideration of necessary test adaptations. This symposium presents results from analyses of the HCAP data in the US, England, Mexico, South Africa, China, and India, highlighting cross-national differences in later-life cognition identified using the HCAP data, and presenting key methodological concerns for cross-national comparisons of cognitive aging. First, Zhang will present findings comparing education gradients in later-life cognitive function across countries. Next, Cho will present longitudinal data comparing the relationships between short-term changes in household wealth in later-life and subsequent cognitive function across countries. Third, Avila-Rieger will present findings comparing sex/gender disparities in later-life cognitive function in the US and India and how they differ by education. Finally, Nichols will conclude the session by discussing differences in the measurement of cognition for the assessment of dementia across countries and implications for data interpretation and the design of future instruments.

